# Identifying a subtype of Alzheimer’s disease characterised by predominant right focal cortical atrophy

**DOI:** 10.1038/s41598-020-64180-4

**Published:** 2020-04-29

**Authors:** Ko Woon Kim, Seongbeom Park, Hyunjin Jo, Soo Hyun Cho, Seung Joo Kim, Yeshin Kim, Hyemin Jang, Duk L. Na, Sang Won Seo, Hee Jin Kim

**Affiliations:** 1 Department of Neurology, Sungkyunkwan University School of Medicine, Samsung Medical Center, Seoul, Korea; 2 Department of Neurology, Jeonbuk National University Medical School & Hospital, Jeonju, Korea; 3 Research Institute of Clinical Medicine of Jeonbuk National University, Jeonju, Korea; 4 Biomedical Institute of Jeonbuk National University Hospital, Jeonju, Korea; 5 Neuroscience Center, Samsung Medical Center, Seoul, Korea; 6 Samsung Alzheimer Research Center, Samsung Medical Center, Seoul, Korea; 7 Department of Neurology, Chonnam National University Hospital, Gwangju, Korea; 8 Department of Neurology, Gyeongsang National University School of Medicine and Gyeongsang National University Changwon Hospital, Changwon, Korea; 9 Department of Neurology, Kangwon National University Hospital, Kangwon National University College of Medicine, Chuncheon, Korea; 10 Department of Health Sciences and Technology, SAIHST, Sungkyunkwan University, Seoul, Korea; 11 Department of Digital Health, SAIHST, Sungkyunkwan University, Seoul, Korea

**Keywords:** Alzheimer's disease, Alzheimer's disease

## Abstract

We aimed to identify an Alzheimer’s disease (AD) subtype with right predominant focal atrophy. We recruited 17 amyloid PET positive logopenic variant primary progressive aphasia (lvPPA) and 226 amyloid PET positive AD patients. To identify AD with right focal atrophy (Rt-AD), we selected cortical areas that showed more atrophy in lvPPA than in AD and calculated an asymmetry index (AI) for this area in each individual. Using a receiver operating characteristic curve, we found that the optimal AI cut-off to discriminate lvPPA from AD was −3.1 (mean AI – 1.00 standard deviation) (sensitivity 88.2, specificity 89.8). We identified 32 Rt-AD patients whose AI was above mean AI + 1.00 standard deviation, 38 Lt-AD patients whose AI was lower than mean AI − 1.00 standard deviation, and 173 Symmetric-AD patients whose AI was within mean AI ± 1.00 standard deviation. We characterized clinical and cognitive profiles of Rt-AD patients by comparing with those of Lt-AD and Symmetric-AD patients. Compared to Symmetric-AD patients, Rt-AD patients had asymmetric focal atrophy in the right temporoparietal area and showed poor performance on visuospatial function testing (*p* = 0.009). Our findings suggested that there is an AD variant characterized by right focal atrophy and visuospatial dysfunction.

## Introduction

Alzheimer’s disease (AD) patients typically have bilaterally symmetric atrophy in the temporoparietal area and present with memory impairment^1^. However, three atypical AD variants are characterized by progressive focal atrophy in the anterior, posterior, and left cortex with symptoms matched to the affected area. AD patients with anterior focal atrophy show behavioral symptoms and are clinically diagnosed with frontal variant AD; those with posterior focal atrophy show dysfunction in visuospatial processing and are clinically diagnosed with posterior cortical atrophy (PCA); and those with left focal atrophy show conduction aphasia and are clinically diagnosed with logopenic variant primary progressive aphasia (lvPPA)^2–4^. Previous studies have suggested that an additional AD subtype with asymmetric atrophy on the right hemisphere might also exist^5–8^. However, little is known about whether an AD variant with right focal atrophy exists. Furthermore, the clinical presentation of this potential variant is unknown.

To identify an AD variant with right focal atrophy we used an anatomical approach because these patients may not be recognized by clinical phenotype alone. The dominant hemisphere (usually the left) is almost exclusively responsible for language while the non-dominant hemisphere (usually the right) is responsible for visuospatial function and attention. However, several studies have shown that visuospatial function or attention are partially influenced by the left hemisphere^9,10^. Thus, a focal defect in the left hemisphere may result in aphasia, which is readily recognized; whereas a focal defect in the right hemisphere may be compensated by the left hemisphere and not have a distinct phenotype.

In this study, we aimed to identify an AD variant with right focal atrophy (Rt-AD) among amyloid PET-positive AD patients. We assumed that the cortical atrophy pattern of this particular subtype would mirror the atrophy pattern of lvPPA, which has asymmetric atrophy in the left temporoparietal area^11^. We identified Rt-AD patients by using a cortical thinning asymmetric index (AI). We then investigated whether an AD variant with right focal atrophy had clinical symptoms matched to the affected area.

## Results

### Demographics

We retrospectively collected data from 226 patients with amyloid PET-positive AD and 17 patients with amyloid PET-positive lvPPA. Of the 243 patients, 32 patients were selected as Rt-AD, 38 patients were selected as Lt-AD, and 173 patients were selected as symmetric (Sym)-AD. Age, sex, education, and CDR score did not differ significantly among Rt-AD, Sym-AD, and Lt-AD (Table 1).

**Table 1:** Participant demographics.

	Rt-AD	Sym-AD	Lt-AD	Bonferroni *post hoc*		
				p Rt-AD vs Symmetric-AD	p Rt-AD vs Lt-AD	p Sym-AD vs Lt-AD
**N**	32	173	38			
**Age**	65 ± 9	68 ± 11	68 ± 7	1.000	1.000	0.909
**Gender F:M**	22:10	94:79	21:17	0.552	0.978	1.000
**Education**	12 ± 4	12 ± 5	12 ± 5	1.000	1.000	0.900
**APOE4 carrier**	10 (36%)	90 (56%)	13 (39%)	0.196	1.000	0.270
**Vascular risk factors**						
Hypertension	14 (44%)	70 (41%)	16 (42%)	1.000	1.000	1.000
Diabetes	1 (3%)	28 (16%)	5 (13%)	0.164	0.626	1.000
Hyperlipidemia	10 (31%)	45 (26%)	4 (37%)	1.000	1.000	0.534
**Handedness**						
Left-handed	0 (0%)	3 (2%)	1 (3%)	1.000	1.000	1.000

AD, Alzheimer’s disease; aMCI, amnestic mild cognitive impairment; Lt-AD, AD with left focal atrophy; Rt-AD, AD with right focal atrophy; Sym-AD, AD with symmetric atrophy

### Cortical thickness

**Figure 1:**
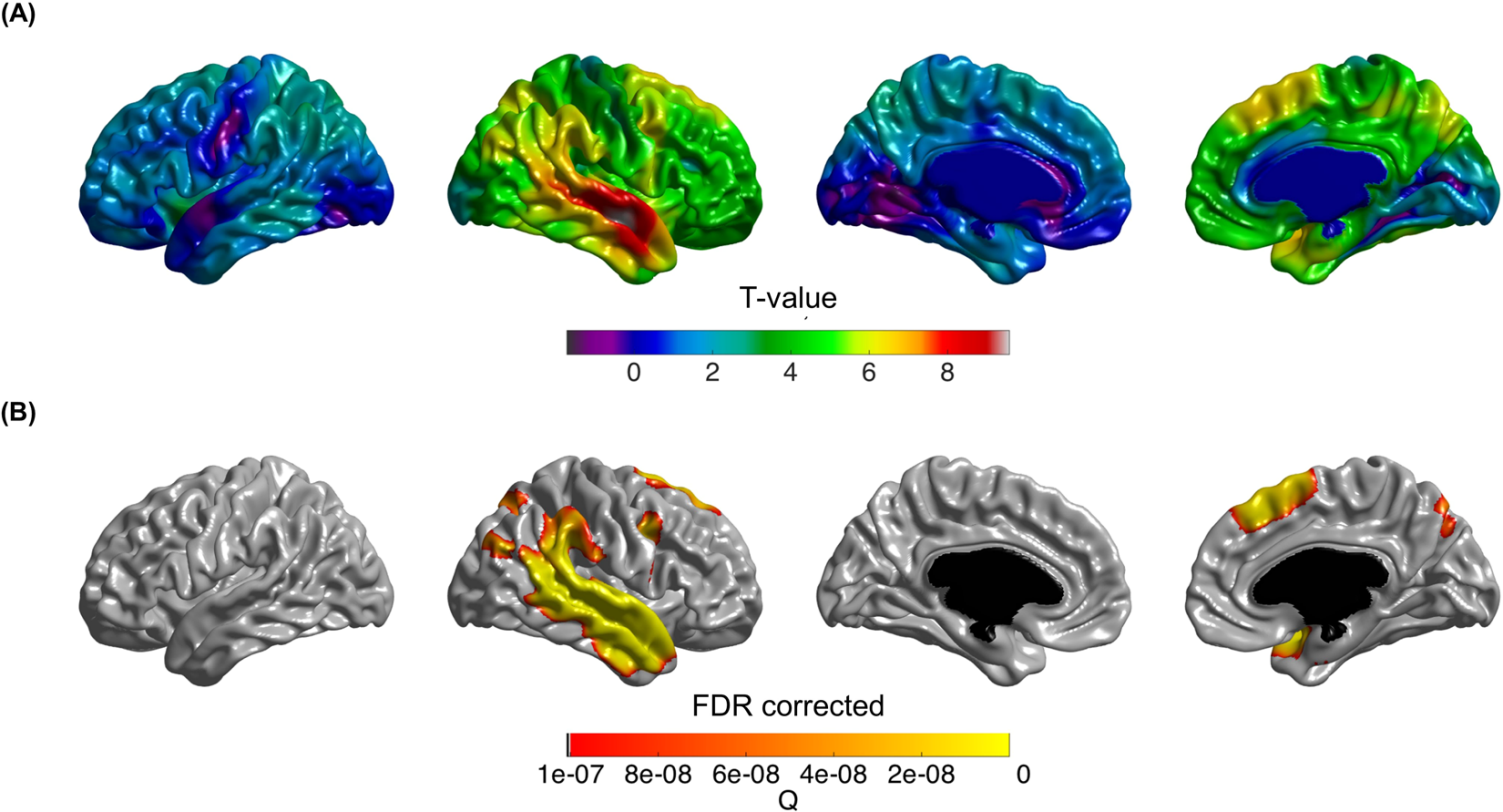
Cortical areas that showed more atrophy in Rt-AD patients compared to Sym- AD patients. Rt-AD group showed significant cortical thinning in the right temporoparietal area compared to Sym-AD patients as shown in t-value (**A**) and FDR corrected, q < 1.0E-7 (**B**). AD, Alzheimer’s disease; AI, Asymmetry index; Rt-AD, AD with right focal atrophy; Sym-AD, AD with symmetric atrophy; SD, standard deviation.

After we defined Rt-AD based on the AI of the superior temporal area (atrophied area of lvPPA compared to AD), we evaluated the overall atrophy pattern of Rt-AD patients. The Rt-AD group had significant cortical thinning in the right temporoparietal area compared to Sym-AD patients (Fig. 1) (*p* < 1.0E-7, FDR corrected).

We performed additional analysis by defining Rt^hemi^-AD based on the AI of the whole hemisphere. Compared to Sym^hemi^-AD patients, Rt^hemi^-AD patients showed cortical thinning in the right temporoparietal area, which was almost the same region shown in Fig. 1 (Supplementary Figure 1).

### Neuropsychological test

Rt-AD patients showed poor performance on visuospatial function testing compared to Sym-AD patients (*p* = 0.013). Rt-AD patients showed poor performance on visual memory testing compared to Sym-AD (*p* = 0.032) and Lt-AD patients (*p* = 0.008). Lt-AD patients showed poor performance on language testing (*p* = 0.002), calculation testing (*p* = 0.024), and frontal/executive function testing (COWAT animal, *p* = 0.004) compared to Sym-AD (Table 2).

**Table 2:** Cognitive profiles of Alzheimer’s disease subtype.

	Rt-AD (n = 32)	Sym-AD (n = 173)	Lt-AD (n = 38)	*p*		
				Rt-AD vs Sym-AD	Rt-AD vs Lt-AD	Sym-AD vs Lt-AD
**Attention**						
Backward digit span	−1.6 ± 1.4	−1.1 ± 1.4	−1.3 ± 1.0	0.517	1.000	1.000
Letter cancellation, abnormal	7 (22%)	26 (15%)	7 (18%)	1.000	1.000	1.000
**Language**						
K-BNT	−2.9 ± 2.7	−2.5 ± 2.1	−5.1 ± 4.3	0.825	0.089	**0.002**
Calculation, abnormal	23 (72%)	101 (62%)	32 (84%)	0.968	0.754	**0.024**
**Visuospatial function**						
RCFT: copying	−8.2 ± 6.7	−4.6 ± 5.6	−4.3 ± 5.3	**0.013**	0.052	1.000
**Memory**						
SVLT: delayed recall	−2.8 ± 0.8	−2.8 ± 1.1	−2.6 ± 0.8	0.927	0.186	0.926
RCFT: delayed recall	−2.6 ± 0.6	−2.3 ± 0.8	−2.1 ± 0.9	**0.032**	**0.008**	0.389
**Frontal/executive functions**						
COWAT animal	−2.0 ± 1.2	−1.7 ± 1.0	−2.5 ± 1.4	0.559	0.235	**0.004**
COWAT phonemic	−1.4 ± 1.2	−1.2 ± 1.1	−1.8 ± 1.1	1.000	0.874	0.113
Stroop test: color	−3.2 ± 2.2	−3.0 ± 2.0	−3.4 ± 1.6	1.000	1.000	0.724
**MMSE**	−8.2 ± 5.0	−5.6 ± 3.9	−8.8 ± 4.7	**0.015**	1.000	**0.001**
**CDR**	1.2 ± 0.8	1.0 ± 0.5	1.0 ± 0.5	0.898	1.000	1.000
**GDepS**	5.1 ± 4.5	4.0 ± 3.4	2.4 ± 2.3	1.000	1.000	1.000

AD, Alzheimer’s disease; CDR, Clinical Dementia Rating; COWAT, Controlled Oral Word Association Test; GDepS, Geriatric Depression Score; K-BNT, Korean version of the Boston Naming Test; lvPPA, logopenic variant primary progressive aphasia; RCFT, Rey-Osterrieth complex figure test; Rt-AD, AD with right focal atrophy; SVLT, Seoul verbal learning test; Sym-AD, AD with symmetric atrophy; MMSE, Mini-mental State Examination.

## Discussion

In this study, we identified AD patients with right focal atrophy, so called Rt-AD, based on the hypothesis that this subtype might be the counterpart to lvPPA. Additionally, we investigated the overall cortical atrophy pattern and neuropsychological characteristics of Rt-AD patients. Our main findings were that there are AD patients who have asymmetric right focal atrophy in the temporoparietal area, and these patients had worse visuospatial function compared to Sym-AD patients.

We identified 32 (14.2%) Rt-AD patients among 226 amyloid-positive AD patients. The Rt-AD patients had asymmetric focal atrophy in the right temporoparietal area. AD is a heterogeneous disease with various subtypes. Each subtype has its unique regions that are vulnerable to neurodegeneration^8,12^. Recent neuroimaging studies clustered AD into 3–5 anatomical subtypes based on cortical atrophy patterns^13,14^. However, a clustering approach might be weak for capturing a specific subtype. Therefore, we searched for Rt-AD patients by using AI of cortical thickness based on the hypothesis that AD with right focal atrophy might be the counterpart of lvPPA.

Our finding that Rt-AD patients had focal atrophy in the temporoparietal area was supported by additional analysis. Instead of calculating AI in the atrophied area of lvPPA, we used the whole hemisphere to calculate AI^hemi^ and defined Rt^hemi^-AD patients based on AI^hemi^ which is strictly anatomical approach. Compared to Sym^hemi^-AD patients, Rt^hemi^-AD patients showed asymmetric focal atrophy in the right temporoparietal area, the same area we found in Rt-AD patients. Furthermore, the atrophied area of lvPPA patients (the left superior temporal area) mirrored that of Rt^hemi^-AD or Rt-AD patients (the right superior temporal area). This suggests that the temporoparietal area might be a unique region vulnerable to neurodegeneration in AD patients with asymmetric focal atrophy.

AD with right focal atrophy may not have been recognized as a clinical syndrome for several reasons. Right hemisphere function involves a diffuse area. The non-dominant hemisphere (right hemisphere in 95% of right handed people and 70% of left handed people^15^) is responsible for visuospatial function, attention, and emotion while the dominant hemisphere is responsible for language and praxis. Because visuospatial function, attention, and emotion are processed by a diffuse bilateral area, a focal defect in the right hemisphere may be partially compensated by the left hemisphere or other areas in the right hemisphere^9,10^. On the other hand, focal lesions in the left hemisphere may cause a profound deficit in language since language is almost exclusively localized to the left inferior frontal, perisylvian, and posterior temporal areas. This is why invasive procedures (ex. ventricular shunts) are more likely to be done on the right hemisphere^16^. Additionally, a subtle language deficit may well be recognized as it causes inconvenience in daily life whereas subtle deficit in visuospatial function, attention, or emotion may not be easily recognized. The most common initial symptom of our Rt-AD patients was episodic memory impairment 50.0% (16/32), followed by both visuospatial dysfunction and episodic memory impairment 28.1% (9/32). We speculate that a focal defect in the right hemisphere in Rt-AD patients might have been compensated by other areas or that a subtle deficit might have been unrecognized.

Although the Rt-AD subtype has not been recognized as a clinical subtype, a group-wise comparison showed that the Rt-AD group had more severe dysfunction in right hemisphere activities, including visuospatial dysfunction and visual memory impairment, compared to Sym-AD patients. Consistent with our study, a previous study showed that visuospatial dysfunction was associated with predominant pathologic changes in the right hemisphere and right dominant atrophy^8^. Clinically defined, rare AD variants such as frontal variant AD, PCA, and lvPPA each have unique focal atrophy in the frontal, posterior, and left cortex, respectively, that matches clinical symptoms^2,3^. Likewise, anatomically defined Rt-AD patients also had visuospatial dysfunction and visual memory impairment that corresponded to cortical thinning in the right temporoparietal area.

Our study has several limitations. First, we searched for the Rt-AD subtype among amyloid-positive AD patients and did not include other amyloid-positive dementia syndromes. Corticobasal syndrome (CBS) or behavioral variant frontotemporal dementia (bvFTD) may also have right asymmetric atrophy with AD as the underlying pathology^17^. Further studies that include other dementia syndromes with AD biomarkers are needed to find the true clinical characteristics of Rt-AD. Second, although our patients were all amyloid PET-positive, we needed pathologic confirmation. Finally, our results need to be replicated with a larger sample.

In conclusion, our study is noteworthy in that we identified an AD variant with right focal atrophy among well-characterized amyloid-positive AD patients. Our findings suggest that there is an AD variant characterized by right focal atrophy and visuospatial dysfunction. Identifying the distinct patterns of structural and neuropsychological characteristics of Rt-AD could provide new insights into regions vulnerable to neurodegeneration in the AD spectrum.

## Methods

### Participants

We retrospectively collected data from 226 patients with amyloid PET-positive AD and 17 patients with amyloid PET-positive lvPPA. Experienced neurologists evaluated the participants based on their clinical symptoms and reviewed medical histories, neuropsychological test results, MRI, amyloid PET, and laboratory tests. AD patients met the research criteria for probable AD dementia with evidence of the AD pathophysiological process proposed by the National Institute of Neurological and Communicative Disorders and Stroke and the Alzheimer’s Disease and Related Disorders Association (NINCDS-ADRDA)^1^. lvPPA patients met current consensus criteria for lvPPA^18^. Participants with other structural lesions, such as territorial infarction, intracranial hemorrhage, brain tumor, hydrocephalus, or severe white matter hyperintensities (WMH), observed on brain MRI were excluded. Severe WMH was defined as a periventricular WMH ≥ 10 mm or a deep WMH ≥ 25 mm as modified from the Fazekas ischemia criteria^19^.

### Standard protocol approvals, registrations, and patient consents

The Institutional Review Board at Samsung Medical Center approved our study and waived the need for informed consent as part of our study approval since we used retrospective de-identified data collected during health exam visits. In addition, all methods were carried out in accordance with the approved guidelines.

### Neuropsychological assessments

All patients underwent a standardized neuropsychological battery called the Seoul Neuropsychological Screening Battery (SNSB)^20,21^. The SNSB consists of tests for attention, language, calculation, visuospatial, memory, frontal-executive function, and tests for general cognition such as the Mini-mental State Examination (MMSE) and Clinical Dementia Rating (CDR). Attention was assessed with the backward digit span and letter cancellation tests; language was assessed with the Korean version of the Boston Naming Test (K-BNT); calculation was assessed with addition, subtraction, multiplication, and division; visuospatial function was assessed with the Rey-Osterrieth Complex Figure Test (RCFT); memory function was assessed with delayed recall of the Seoul Verbal Learning Test (SVLT) and RCFT; and frontal-executive function was assessed with the Controlled Oral Word Association Test (COWAT) phonemic fluency test and the Stroop color reading test. Each score was converted into a standardized Z-score based on age- and education-adjusted norms.

### Brain MRI scans

All patients underwent brain MRI including fluid-attenuated inversion recovery (FLAIR) and three-dimensional (3D) T1 imaging at Samsung Medical Center in a 3.0 T MRI scanner (Philips 3.0 T Achieva; Best, the Netherlands).

### Amyloid PET scan

Amyloid accumulation in the brain was assessed with amyloid PET scans: ^11^C-Pittsburgh compound B (PiB) PET (n = 67), ^18^F- florbetaben PET (n = 173), ^18^F-florbetapir PET (n = 2), or ^18^F-flutemetamol PET (n = 1). Amyloid PET scans were obtained with a Discovery STe PET/CT scanner (GE Medical Systems, Milwaukee, WI, USA) at Samsung Medical Center. Amyloid PET positivity was interpreted based on previously reported guidelines for each ligand. PiB PET positivity was defined as a global ^11^C- PiB uptake ratio more than two SD from the mean of normal controls (PiB retention ratio ≥ 1.5)^22^. ^18^F-florbetaben PET^23^, ^18^F-florbetapir PET^24^, and ^18^F-flutemetamol PET^25^ positivity were defined based on visual assessment systems.

### Cortical thickness analysis

The standard Montreal Neurological Institute (MNI) anatomical pipeline was used to extract cortical thickness. Native MRI images were registered to a standardized stereotaxic space with a linear transformation^26^. The N3 algorithm was used to correct intensity nonuniformities due to inhomogeneities in the magnetic field^27^. The registered and corrected images were classified into white matter, gray matter, cerebrospinal fluid, and background with a 3D stereotaxic brain mask and the Intensity-Normalized Stereotaxic Environment for Classification of Tissues (INSECT) algorithm^28^. The inner and outer cortical surfaces were automatically extracted with the Constrained Laplacian-based Automated Segmentation with Proximities (CLASP) algorithm^29^.

The cortical thickness was defined as the Euclidean distance between the linked vertices of the inner and outer surfaces, after applying an inverse transformation matrix to cortical surfaces and reconstructing them in the native space^29,30^.

### Identifying AD variants with right focal atrophy

**Figure 2:**
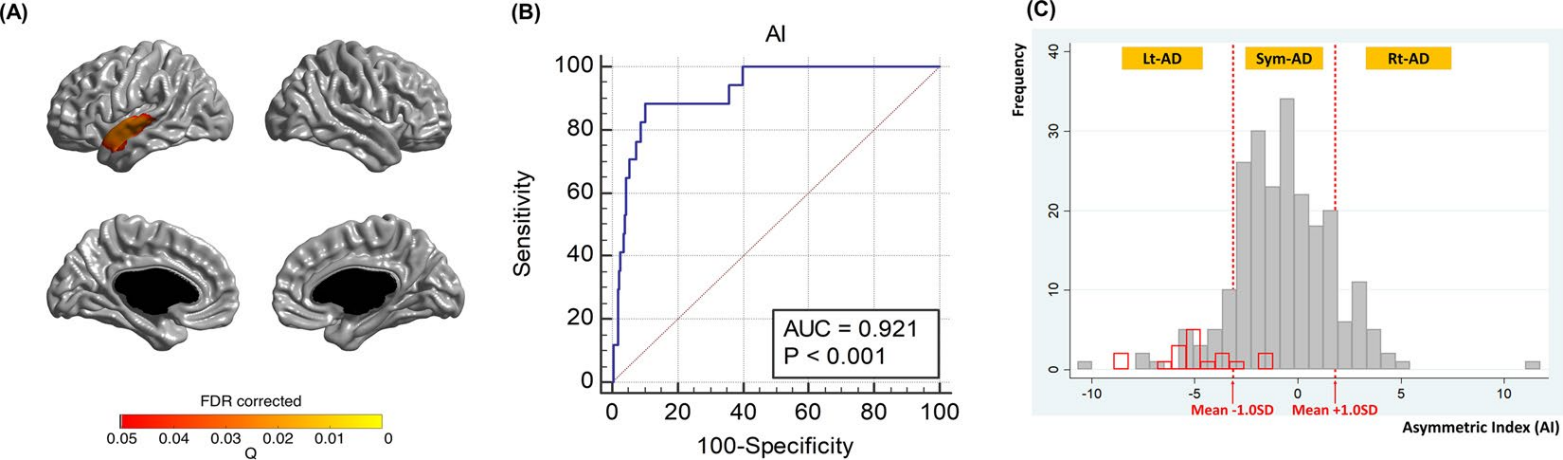
Identifying Rt-AD patients. (**A**) We selected the cortical area that showed more atrophy in lvPPA compared to AD and defined that area as the region of interest (ROI) (q < 0.05 FDR corrected). (**B**) We calculated an asymmetry index (AI) for the ROI in each participant. The optimal AI cut-off to discriminate lvPPA from AD was identified by using a receiver operating characteristic curve (sensitivity 88.2, specificity 89.8, area under curve 0.921, p < 0.001). This cut-off value (−3.1377) corresponds to the mean AI – 1.00 SD. (**C**) Finally, we identified 32 Rt-AD patients by selecting patients who had an AI value greater than the mean AI + 1.00 SD (1.8112). We also identified 38 Lt-AD patients by selecting patients who had an AI value lower than the mean AI − 1.00 SD (−3.1377) and identified 173 Sym-AD patients who had an AI value within mean AI ± 1.00 SD (between −3.1377 and 1.8112). Red box indicates lvPPA and gray box indicates AD. AD, Alzheimer’s disease; AI, Asymmetry index; lvPPA, logopenic variant primary progressive aphasia; Lt-AD, AD with left focal atrophy; Rt-AD, AD with right focal atrophy; Sym-AD, AD with symmetric atrophy; SD, standard deviation.

Rt-AD patients were identified based on the assumption that the Rt-AD cortical atrophy pattern would be the counterpart of lvPPA. In other words, we selected patients whose cortical atrophy pattern mirrored that of lvPPA. To identify Rt-AD patients, we began by selecting a cortical area that showed more atrophy in lvPPA compared to AD. That area, the left superior temporal area, was defined as the region of interest (ROI) (Fig. 2A). We then calculated an asymmetry index (AI)^31^ for the ROI in each participant as follows:

$$AI=\frac{L-R}{L+R}\times 100$$

R and L represent mean cortical thicknesses of corresponding voxels from the right and left ROI, respectively. The optimal AI cut-off to discriminate lvPPA from AD was identified by using a receiver operating characteristic curve (sensitivity 88.2, specificity 89.8) (Fig. 2B). This cut-off value (−3.1377) corresponds to the mean AI – 1.00 SD. Finally, we identified 32 Rt-AD patients by selecting patients who had an AI value greater than the mean AI + 1.00 standard deviation (1.8112) (Fig. 2C). To make a fair comparison, we identified 38 Lt-AD patients by selecting patients who had an AI value lower than the mean AI − 1.00 SD (−3.1377) and identified 173 Sym-AD patients whose AI was within mean AI ± 1.00 SD (between −3.1377 and 1.8112).

We performed additional analysis to identify AD patients with right focal atrophy using strictly anatomical method. Instead of using ROI that was defined based on the atrophied area of lvPPA, we used the whole hemisphere to calculate AI^hemi^.

$$AI(hemi)=\frac{L(hemi)-R(hemi)}{L(hemi)+R(hemi)}\times 100$$

R(hemi) and L(hemi) represent mean cortical thickness of right and left hemisphere, respectively. Mean hemispheric AI of all patients (lvPPA and AD) was −1.1478. We arbitrarily defined Rt^hemi^-AD when AI^hemi^ value of the patient was greater than the mean AI^hemi^ + 1.00 SD (1.7872) (n = 32). Likewise, we defined Lt^hemi^-AD when AI^hemi^ value of the patient was lower than the mean AI^hemi^ − 1.00 SD (−4.0827) (n = 30), and defined Sym^hemi^-AD when AI^hemi^ value of the patient was within mean AI^hemi^ ± 1.00 SD (n = 181) (Supplementary Figure 2).

### Statistics

To assess demographics among the three groups, we used the chi-square test or Fisher’s exact test for categorical variables followed by Bonferroni post hoc analysis. We used the Kruskal-Wallis test for continuous variables because variables did not follow a normal distribution. We performed *post hoc* comparisons with the Mann–Whitney U-test with a Bonferroni correction.

To investigate differences in cognitive profiles among the three groups, we used the Kruskal-Wallis test because a Kolmogorov-Smirnov test indicated that the Z-score of the cognitive profiles did not follow a normal distribution. We performed *post hoc* comparisons with the Mann–Whitney U-test with a Bonferroni correction.

Statistical comparisons for cortical thickness between groups were performed on a voxel-by-voxel basis with t-statistics. Multiple comparisons were corrected using the false discovery rate (FDR) procedure (*p* < 0.05). We used the SurfStat package created by Dr. Keith Worsley (http://www.math.mcgill.ca/keith/surfstat).

## Supplementary information

Supplementary Information.

## Data Availability

The datasets generated during and/or analyzed during the current study are available from the corresponding author on reasonable request.
