# Supplementary material for: Identifying a subtype of Alzheimer’s disease characterised by predominant right focal cortical atrophy
Source: Sci Rep. 2020 Apr 29;10:7256. doi: 10.1038/s41598-020-64180-4 (PMC7190862; doi:10.1038/s41598-020-64180-4)
Supplement: Supplementary file 1 — Supplementary Information. [file 41598_2020_64180_MOESM1_ESM.docx]

**Identifying a subtype of Alzheimer’s disease**

**characterised by predominant right focal cortical atrophy**

Ko Woon Kim, MD, PhD,^1,2,3,4#^ Seongbeom Park, MS,^1,5,6#^ Hyunjin Jo, MD,^1,5^ Soo Hyun Cho, MD, PhD,^1,7^, Seung Joo Kim, MD,^1,8^, Yeshin Kim, MD, ^1,9^ Hyemin Jang, MD,^1,5,6^ Duk L Na, MD, PhD,^1,5,6,10,12^ Sang Won Seo, MD, PhD,^1,5,6,11,12^ Hee Jin Kim, MD, PhD^1,5,6,10,12^*

^1^Department of Neurology, Sungkyunkwan University School of Medicine, Samsung Medical Center, Seoul, Korea

^2^Department of Neurology, Jeonbuk National University Medical School & Hospital, Jeonju, Korea

^3^Research Institute of Clinical Medicine of Jeonbuk National University

^4^Biomedical Institute of Jeonbuk National University Hospital

^5^Neuroscience Center, Samsung Medical Center, Seoul, Korea

^6^Samsung Alzheimer Research Center, Samsung Medical Center, Seoul, Korea

^7^Department of Neurology, Chonnam National University Hospital, Gwangju, Korea

^8^Department of Neurology, Gyeongsang National University School of Medicine and Gyeongsang National University Changwon Hospital, Changwon, Korea

^9^Department of Neurology, Kangwon National University Hospital, Kangwon National University College of Medicine, Chuncheon, Korea

^10^Department of Health Sciences and Technology, SAIHST, Sungkyunkwan University, Seoul, Korea

^11^Department of Clinical Research Design and Evaluation, SAIHST, Sungkyunkwan University, Seoul, Korea

^12^Department of Digital Health, SAIHST, Sungkyunkwan University, Seoul, Korea

**#These two authors contributed equally to this work**

***Corresponding author:**

Hee Jin Kim, MD, PhD

Department of Neurology, Sungkyunkwan University School of Medicine, Samsung Medical

Center, 50 Ilwon-dong, Gangnam-ku, Seoul 06351, Republic of Korea

Phone: +82-2-3410-6147 Fax: +82-2-3410-0052, E-mail: [evekhj@gmail.com](mailto:evekhj@gmail.com)


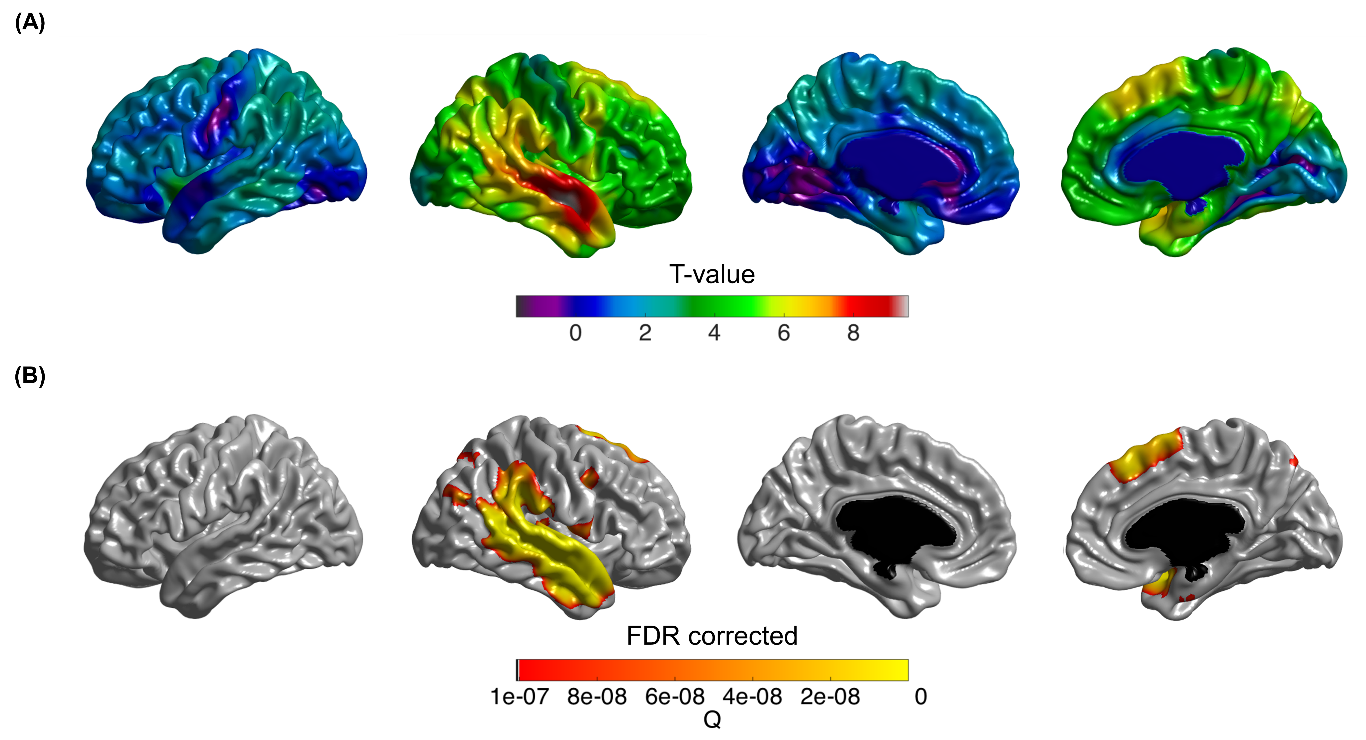


**Supplementary Figure 1**

Cortical areas that showed more atrophy in Rt^hemi^-AD patients compared to Sym^hemi^-AD patients as shown in t-value (A) and FDR corrected, q < 1.0E-7 (B).


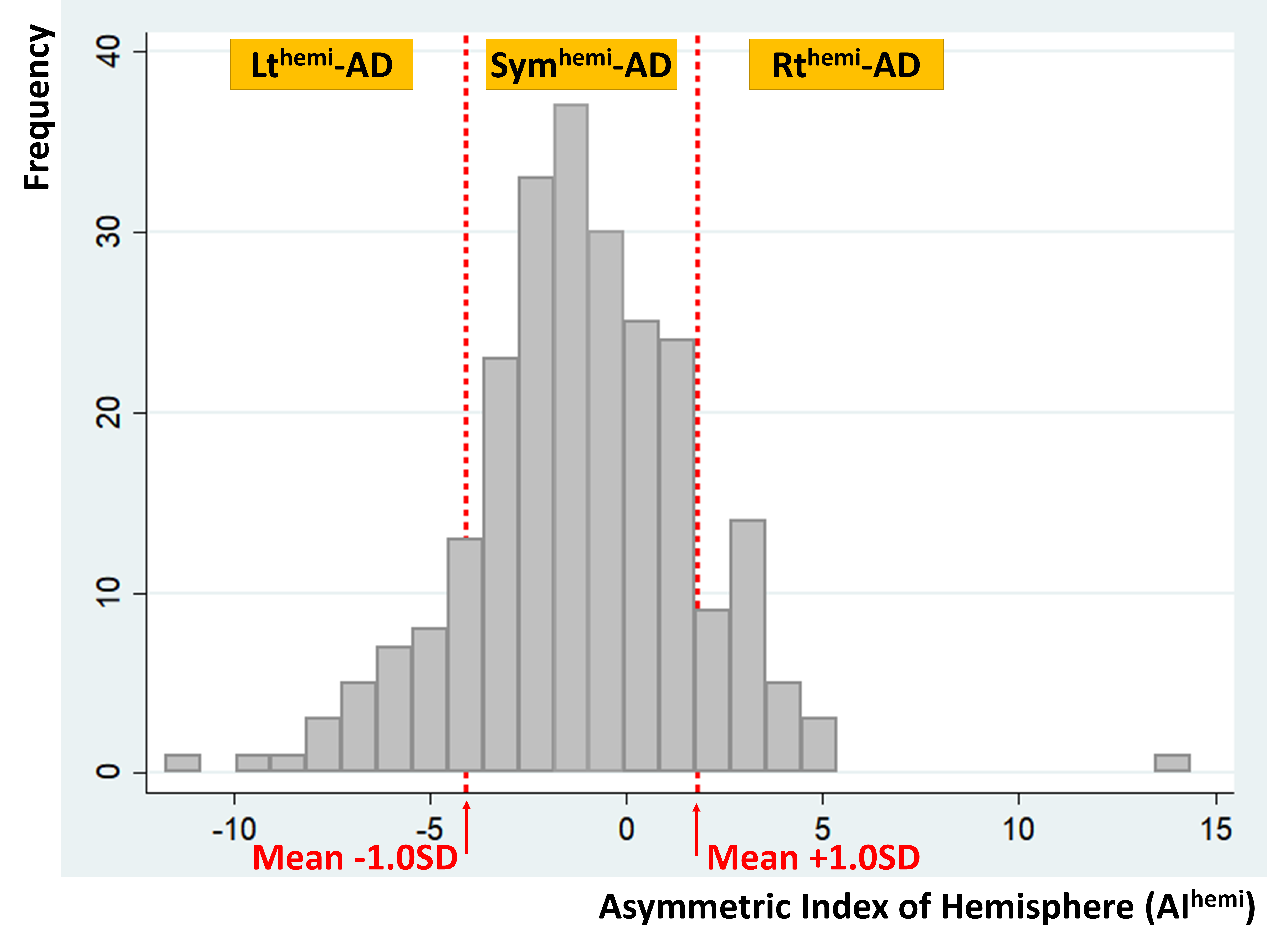


**Supplementary Figure 2.** Identifying Rt^hemi^-AD patients

We used the whole hemisphere to calculate AI^hemi^. We arbitrarily defined Rt^hemi^-AD when AI^hemi^ value of the patient was greater than the mean AI^hemi^ + 1.00 SD (1.7872) (n=32). Likewise, we defined Lt^hemi^-AD when AI^hemi^ value of the patient was lower than the mean AI^hemi^ - 1.00 SD (-4.0827) (n=30), and defined Sym^hemi^-AD when AI^hemi^ value of the patient was within mean AI^hemi^ ± 1.00 SD (n=181)
